# Single German centre experience with patient journey and care-relevant needs in amyloidosis: The German _A_MY-NEED_S_ research and care program

**DOI:** 10.1371/journal.pone.0297182

**Published:** 2024-05-20

**Authors:** Sandra Michaela Ihne-Schubert, Maria Leberzammer, Marcel Weidgans, Stefan Frantz, Hermann Einsele, Stefan Knop, Torben Schubert, Tanja Bratan, Stefan Störk, Silke Neuderth

**Affiliations:** 1 Interdisciplinary Amyloidosis Centre of Northern Bavaria, University Hospital Würzburg, Würzburg, Germany; 2 Department of Internal Medicine II, Hematology, University Hospital Würzburg, Würzburg, Germany; 3 CIRCLE - Centre for Innovation Research, Lund University, Lund, Sweden; 4 Department of Internal Medicine IV, University Hospital Gießen and Marburg, Gießen, Germany; 5 Comprehensive Heart Failure Centre (CHFC) Würzburg, University and University Hospital Würzburg, Würzburg, Germany; 6 Department of Internal Medicine I, Cardiology, University Hospital Würzburg, Würzburg, Germany; 7 Department of Internal Medicine 5, Klinikum Nürnberg Nord, Nürnberg, Germany; 8 Competence Center Innovation and Knowledge Economy, Fraunhofer Institute for Systems and Innovation Research ISI, Karlsruhe, Germany; 9 Competence Center Emerging Technologies, Business Unit “Innovations in the Health System”, Fraunhofer Institute for Systems and Innovation Research ISI, Karlsruhe, Germany; 10 Institute for Applied Social Sciences (IFAS) of the Technical University of Applied Sciences Würzburg-Schweinfurt (THWS), Würzburg, Germany; Scuola Superiore Sant’Anna, ITALY

## Abstract

**Background:**

Amyloidosis is a rare multi-system disorder associated with frequently delayed diagnosis, enormous disease burden and psychosocial distress.

**Methods:**

Systematic assessment of needs was performed by a subtype-spanning questionnaire-based survey within the _A_MY-NEED_S_ research and care program.

**Results:**

118 patients with proven amyloidosis (62.7% ATTR, 22.0% AL, 15.3% other forms) were included in August 2020 until February 2021 (mean age 71.2 ±11.3 years; 30% women). The median diagnostic delay between onset of symptoms and diagnosis was 9.0 (range: 2.5; 33.0) months. Local health care providers (HCPs) play a central role on the way to diagnosis. Diagnosis itself typically requires a clinical but not necessarily a university setting. In the treatment phase, the focus moves to the amyloidosis centre as primary contact and coordinator, with general practitioners (GPs) acting predominantly as a contact point in crisis and link to additional services. About half of patients reported impaired quality of life and one third suffering from anxiety and depressed mood, respectively. The majority of patients talk about their concerns with close caregivers and local HCPs. Advance care planning is a relevant, yet insufficiently met need.

**Conclusion:**

The journey of patients with amyloidotic disease, their contact partners and needs at different stages were characterized in detail within the German health care system. An amyloidosis-specific care concept has to master the multitude of interfaces connecting the numerous treatment providers involved with the amyloidosis centre and GPs as key players. Telemedical approaches could be a promising and well-accepted option allowing optimal coordination and communication.

## Introduction

Amyloidosis is a rare systemic disease in which protein deposits form insoluble fibrils in the tissues of various organs with consecutive organ dysfunction [1, 2]. The clinical phenotype including symptom severity, speed of progression, and prognosis, heavily depends on the causative protein, pattern and severity of organ involvement. Transthyretin (ATTR) amyloidosis occurring as hereditary (ATTRv) or age-related (ATTRwt) forms and light chain (AL) amyloidosis are the most frequent subtypes, whereas AA amyloidosis, localized amyloidosis, and rare forms such as ApoC2 amyloidosis are less common. Due to non-specific clinical symptoms in the early stages of the disease, diagnosis is frequently delayed and consequently recognized in advanced stages [3–5]. The median diagnostic delay is estimated to be 2.7 years for AL, 3.4 years for ATTRwt and 2.6 years for ATTRv amyloidosis [3, 5]. The importance and the benefit of early diagnosis has been demonstrated for both AL and ATTR amyloidosis [6–8].

However, a considerable diagnostic delay is typical for rare diseases in general, as they are often considered as differential diagnosis very late in the diagnostic process. In recent years, numerous campaigns, foundations and the establishment of centres for the interdisciplinary treatment and research of rare diseases have attempted to raise awareness on several rare diseases. An important milestone in this context is the founding of the National Action Alliance for People with Rare Diseases (NAMSE) in 2010 with the goal of improving the life situation of every single person with a rare disease. With regard to amyloidosis, there has only been one amyloidosis centre in Germany since 2008 in Heidelberg, the second was just founded in 2018 in Würzburg. However, the establishment of DPD scintigraphy as a non-invasive diagnostic tool for ATTR amyloidosis [9], increasing availability of causal therapeutic options [10–17] and increased awareness of amyloidosis due to numerous activities on the part of treatment providers and the pharmaceutical industry resulted in a marked improvement of the care situation as already shown for Great Britain [8]. In parallel, a couple of additional centres have been built up throughout Germany in the recent past.

Due to its complex nature, diagnosis (and differential diagnosis) of amyloidosis requires well-coordinated interdisciplinary algorithms. Accordingly, effective and timely treatment mandates easily accessible integrative interdisciplinary care structures that are able to address the special features of this disease. For this reason and due to high time and cost intensity the amyloidosis-specific subtype-spanning care structures are primarily university or university-related. Specialized non-university practices or outpatient clinics are usually focused on individual subentities.

In principle, there is a free choice of doctor in the German health care system. Only patients in the general practitioner (GP) model have to consult primarily their GP. Nevertheless, the GP has a key role as the primary contact person for many patients. Accordingly, data from the Federal Association of Statutory Health Insurance Physicians from 2021 show that 81% of patients with statutory health insurance obtained care from a GP in the previous year, half of them exclusively; while 19% of patients only consulted a specialist [18]. Furthermore, data show that while GP appointments are generally available in a timely manner, waiting times for specialist appointments are still over 3 weeks in nearly 30%.

Against this background and known numerous physician contacts on the patient journey [4], another challenge could be the structure of the German health care system itself, and a key figure might be the GP.

Up to now, there are only few studies that tracked the journey of amyloidosis patients, primarily from the United States [3, 19]. Data regarding the situation in Germany are scarce.

To better understand the patient journey with amyloidosis in the German health care system we aimed to

i) outline the patient journey in the German health system
ii) characterize the needs of these patients on their way to diagnosis and in the course of treatment with a focus on infrastructures and resources used
iii) quantify the needs expressed for psychosocial support.

## Methods

The rationale and design of the _A_MY-NEED_S_ research and care program for patients with amyloidosis is described elsewhere [20]. In brief, the AMY-NEEDS study represents a sub-study of the prospective amyloidosis cohort study AmyKoS of the Interdisciplinary Amyloidosis Centre Northern Bavaria. The Ethical Committee of the University of Würzburg approved the study design and protocol of _A_MY-NEED_S_ (#37/19) and of AmyKoS (#48/18). Using a mixed-methods approach, the needs of affected patients, their relatives and their health care providers (HCPs) were systematically assessed with the aim of developing an amyloidosis-specific care concept. First, focus groups were conducted for needs analysis among all three target groups. Their audio-records were analysed qualitatively and content-analytically according to Ruddat et al. 2012 [21] using version 20 of MAXQDA^®^ regarding the following aspects [20]: general important aspects of treatment and care at the amyloidosis centre, special needs in different phases of the disease, desired support from different treatment groups, review of possible helpful factors in the course of treatment, experienced cooperation between the amyloidosis centre, general practitioners (GP) and specialists, additional support options.

Based on these qualitative results [20], a questionnaire to assess patients’ needs was developed (S1 File). This questionnaire contains 12 domains including 165 key questions that were rated (if relevant) using 4-point Likert scales. The domains are summarized in Table 1. The validation of the questionnaire was performed by cognitive interviews of ten patients using the “Think aloud” approach. No relevant problems in the understanding of the questionnaire were identified.

**Table 1:** Domains of the _A_MY-NEED_S_ phase II questionnaire.

	domains	example(s)	number of questions
1	sociodemographic data	*age*, *gender*, *nationality*, *education*, *family status*	15
2	characterization of the patient journey	*type and time point of first complaints*, *contact partners at different stages of the patient journey*	7
3	general health status and need for help, identification of care givers	*Third party support*, *primary care givers*	9
4	general and disease-specific situation from patients’ view	*subtype of amyloidosis*, *impairment of quality of life*	19
5	current patient situation	*current use of care services such as contact with GPs; disease burden*	12
6	weighting of needs on the way to diagnosis, at diagnosis and in the further course of the disease	*acceleration of diagnosis*, *comprehensiveness of diagnostic investigations*	17
7	current degree of implementation at the local amyloidosis centre	*acceleration of diagnosis*, *comprehensiveness of diagnostic investigations*	17
8	allocation of responsibilities on the way to diagnosis, at diagnosis and in the further course of the disease	*primary medical contact*, *provision of information about the suspected disease*	28
9	possibilities for optimization at the interface between the amyloidosis centre and the local HCPs as well as between the amyloidosis centre and the patient.	*Electronic medical file*, *hotline*	17
10	use of and attitude towards telemonitoring	*use of telephone- or video-based telemonitoring*	3
11	communication about concerns and worries	*fear of a relapse*	12
12	coping strategies	*physical or social activities*	8

The questionnaire-based quantitative analysis was performed across the local AmyKoS population and was followed by a survey among their peripheral HCPs.

The presented data refer exclusively to the questionnaire survey of the AmyKoS patients. Inclusion criteria for the questionnaire-based patient survey were the participation in AmyKoS as well as informed consent to _A_MY-NEED_S_ study participation. There were no further exclusion criteria. All AmyKoS participants were asked to participate in 2020 and further newly included AmyKoS participants were consecutively recruited via the outpatient department until February 2021. All participants provided written informed consent prior to any investigation.

The patients were surveyed by telephone for the questionnaire-based survey from August 2020 to February 2021. Responses were documented in paper CRFs during the survey and then pseudonymized using the AmyKoS IDs of the patients and entered into a study-specific database. The reported clinical data refer to the next clinical evaluation in the centre within the framework of the AmyKoS.

A total of 222 patients were asked to participate in the study. 18.9% of the patients primarily refused to participate and additional 8.6% subsequently withdrew consent until the survey, mostly due to a deterioration in their mental or physical condition. 3.2% of the patients were not able to participate due to dementia or insufficient language skills and 1 patient (0.5%) died after inclusion. A total of 153 patients were surveyed. In the analysis reported here, only patients with confirmed amyloidosis were considered (n = 118).

### Data analysis

Data are described using frequency (percent), mean values with standard deviation or median values with ranges. Groups were compared by t-tests.

To estimate the changes in diagnostic delay attributable to the foundation on the amyloidosis centre, quantile regression was used. Quantile regression pioneered by Koenker and Bassett (1978) is methodologically related to linear regression (OLS) but aims at different distribution parameters [22]. While ordinary least squares, however, can be shown to identify the function *E*(*y*│*x*) = *xβ* with *y* being the explained variable, *x* a vector of explanatory variables and *β* a vector of coefficients to be estimated, quantile regression can identify specific quantiles of the *y*:

$$Q_{p}\left(y|x\right)=x\beta^{p}\;\;\;\;\;,\;p\in ]0,1[$$ (1)

The interpretation of the estimated coefficients is the same as in OLS: if a coefficient $\beta_{k}^{p}$ associated with some explanatory variable is positive for, say, the median *Q*_0.5_, it means that the median increases by $\beta_{k}^{p}$ units for every marginal unit increase of *x*_*k*_. It is important to note that the coefficients will generally differ by quantile.

S1 Fig shows the coefficients of the quantile regressions (p = 0.1, 0.2,….0.9) of the diagnostic delay (dependent variable) on a dummy variable which is one for the time when the amyloidosis centre was already founded (2018 and after) and zero before. This specification implies that the coefficients are indicative of the effects of the foundation of the centre on the diagnostic delay.

Statistical tests were carried out using R (version 3.6.3). Quantile regression was performed using STATA^®^ version 14.

## Results

### Characterization of the cohort

The analyzed sample consists of 118 patients, surveyed from August 2020 to February 2021. 70.3% of the participants were male. The average age of the total sample was 71.2 ±11.3 years. 62.7% (n = 74) of patients suffered from ATTR amyloidosis, including 11 patients (9.3%) with the hereditary ATTR (ATTRv) amyloidosis form. 22.0% (n = 26) had a known systemic and 10.2% (n = 12) a localized light chain (AL) amyloidosis. AA amyloidosis was found in 4.2% (n = 5) of patients and ApoC2 amyloidosis was documented in 0.8% (n = 1) of surveyed patients. 74.6% of the patients showed cardiac involvement (CA). Further details are summarized in Table 2.

**Table 2:** Characterization of the analyzed cohort.

Aspect	Results within the cohort	
General & medical aspects		
number of patients	118	
time period	August 2020 to February 2021	
Sex	70.3% men; 29.7% women	
Age	71.2 ± 11.3 years,	
ECOG	median 0 (range 0–4)	
subtype of amyloidosis	ATTR amyloidosis	62.7% (n = 74)
	ATTRv amyloidosis	9.3% (n = 11)
	ATTRwt amyloidosis	53.4% (n = 63)
	systemic AL amyloidosis	22.0% (n = 26)
	localized AL amyloidosis	10.2% (n = 12)
	AA amyloidosis	4.2% (n = 5)
	ApoC2 amyloidosis	0.8% (n = 1)
Comorbidities		
Charlson comorbidity index*	median 2.0** (range 0–11 points)	
Common comorbidities	heart failure***	57.6%
	tumor diseases	20.3%
	lymphomas	13.6%
	kidney diseases	16.9%
	chronic lung diseases	11.9%
	diabetes mellitus	10.2%
Organ involvement		
heart	74.6% overall (AL-CA 65.4%, ATTR-CA 94.6%)	
kidney	19.5% overall (AL-CA 61.5%, ATTR-CA 0%)	
PNS/ANS^#^	17.8% overall (AL-CA 12.5%, ATTR-CA 24.3%)	
GIT^##^	5.9% overall (AL-CA 15.4%, ATTR-CA 0%)	
liver	1.7% overall (AL-CA 3.8%, ATTR-CA 0%)	
Other	15.3% overall (AL-CA 11.5%, ATTR-CA 5.4%)	
Cardiac markers		
NT-proBNP^###^	2292.5 ±3819.4pg/ml (overall)	
high-sensitive troponin	45.1±24.8pg/ ml (overall)	
General and social patient situation		
German citizenship	97.5%	
distance to the centre	21.2% lived in the city or county of the centre	
> 150 km	29.7%	
50–100 km	28.9%	
≤ 50 km	32.2%	
no information	9.3%	
Employment		
old-age	69.5%	
disability pension	11.9%	
still employed	15.3%	
unemployed	2.5%	
average net household income	over 2000 €: 66.1%	
severely disabled person’s pass	59.3%, median grade of 75% (range 30–100%)	
	in addition 1.7% had applied	
Care degree (total)	17.8%	
I (lowest grade)	28.6%	
II	33.3%	
III	23.8%	
IV	9.5%	
V (highest grade)	4.8%	
Need for assistance from third parties		
everyday activities	39.8% overall (ATTR 44.6%, AL 46.2%)	
personal hygiene	59.6% overall (ATTR 66.7%, AL 50.0%)	
household activities	85.1% overall (ATTR 84.8%, AL 83.3%)	
shopping	72.3% overall (ATTR 78.8%, AL 66.7%)	
other activities	10.6% overall (ATTR 12.1%, AL 8.3%)	
Primary supporters		
partners	72.9%	
children or grandchildren	18.6%	
other relatives	3.4%	
friends	2.5%	
No supporters	2.5%	

* Charlson comorbidity index (CCI): 0 = no risk, > 5 = 1-year-mortality risk >85%)

** corresponding to an estimated general 1-year mortality risk of 26%

*** due to amyloidosis

^#^ peripheral and autonomous nervous system

^##^ gastrointestinal tract

^###^ N-terminal pro B-type natriuretic peptide

### Symptoms at diagnosis

The symptoms at diagnosis were highly variable (Table 3). 18.6% of patients described general symptoms such as fatigue, weakness and a decline in performance. Cardiopulmonary symptoms dominated (55.9%). Neurological symptoms were the second most common (24.6%). 19.5% of patients received their diagnosis incidentally. A subtype-specific analysis revealed a frequency of incidental diagnosis among ATTR amyloidosis patients of 20.3% and among systemic AL amyloidosis patients of 7.7%.

**Table 3:** Symptoms at disease onset (from patients’ point of view).

Symptoms	n	%	Definition of symptom	n	%
General symptoms	22	18.6%	drop in performance	10	8.5%
			weakness	7	5.9%
			fatigue	3	2.5%
Cardiopulmonary symptoms	66	55.9%	dyspnea at rest or at exertion	35	29.7%
			peripheral edema	14	11.9%
			cardiac events (e.g. myocardial infarction, cardiac arrest)	3	2.5%
			circulatory distress, syncope, collapse	3	2.5%
			cough	2	1.7%
			pulmonary edema	2	1.7%
Gastrointestinal symptoms	8	6.8%	diarrhea	3	2.5%
Neurological symptoms	29	24.6%	pain	6	5.1%
			dizziness, balance disorders	5	4.2%
			polyneuropathy	4	3.4%
			sensory disturbances	4	3.4%
			migraine	2	1.7%
			vision problems	2	1.7%
Other	12	10.2%	bleeding tendency/bleeding	3	2.5%
			hoarseness	3	2.5%
Laboratory abnormalities	2	1.7%		2	1.7%
Incidental diagnosis	20	16.9%		20	16.9%

Only results > 1% are reported.

### Diagnostic delay and diagnostic pathway

The mean age at diagnosis was 68.0 ±13.1 years. The diagnostic delay was defined as duration between onset of symptoms and primary diagnosis of amyloidosis. It was assessed in the survey and later compared with the medical documentation (if available). 23 participants with incidental diagnosis were excluded from analysis resulting in a data set of 95 patients for this question. In total, 91 of the remaining patients (n = 95) were able to quantify the diagnostic delay with resulting median diagnostic delay of 9.0 months (range 0–120 months). The median subtype-specific delay was 6.0 months (range 0–60, n = 24) for AL and 12.0 months (range 0–120 months, n = 56) for ATTR amyloidosis.

Using the medical documentation as basis, additional 18 of the 95 patients had to be excluded from the analysis due to missing or imprecise data regarding onset of symptoms and/or the establishment of diagnosis. Median subtype-spanning diagnostic delay of the remaining 77 patients was similar: 7.9 months (range 0–120 months); broken up by subtype, delay was 5.9 months (range 1–48 months, n = 21) for AL and 12.0 months (range 0–121.7 months, n = 47) for ATTR amyloidosis.

Within the first month after symptom onset (based on the survey data, Table 4), only 23.2% of ATTR and 8.3% of systemic AL amyloidosis were detected, respectively. In the first year after symptom onset, a total of 51.8% of ATTR and 62.5% of systemic AL amyloidosis patients, had received their diagnosis. 25.0% of ATTR and 12.5% of systemic AL amyloidosis patients had to wait for more than 3 years.

**Table 4:** Diagnostic delay (based on the survey data).

	total cohort			ATTR amyloidosis			AL amyloidosis		
	*number of diagnosed patients*	*percentage of additional diagnosis*	*percentage of diagnosed patients*	*number of diagnosed patients*	*percentage of additional diagnosis*	*percentage of diagnosed patients*	*number of diagnosed patients*	*percentage of additional diagnosis*	*percentage of diagnosed patients*
1st month	18		23.4%	13		**23.2%**	2		**8.3%**
until end of 3^rd^ months	24	7.8%	31.2%	19	10.7%	**33.9%**	5	12.5%	**20.8%**
until end of 6^th^ months	35	14.3%	45.5%	24	8.9%	**42.9%**	13	33.3%	**54.2%**
until end of 9^th^ months	40	6.5%	51.9%	25	1.8%	**44.6%**	15	8.3%	**62.5%**
until end of 1^st^ year	46	7.8%	59.7%	29	7.1%	**51.8%**	15	0.0%	**62.5%**
until end of 2^nd^ year	55	11.7%	71.4%	35	10.7%	**62.5%**	20	20.8%	**83.3%**
until end of 3^rd^ year	63	10.4%	81.8%	42	12.5%	**75.0%**	21	4.2%	**87.5%**
more than 3 years	77	18.2%	100.0%	56	25.0%	**100.0%**	24	12.5%	**100.0%**
included number of patients	77			56			24		

To estimate the effect of the key changes in the recent past, in particular increasing establishment of specialized outpatient departments and amyloidosis centre(s), establishment of non-invasive diagnosis of cardiac ATTR amyloidosis by bone scintigraphy [9], increasing availability of new drugs [10, 12, 13, 16] and growing disease awareness among HCPs, we i) analyzed the diagnostic delay year by year and ii) performed a quantile regression analysis regarding the diagnostic delay depending on the onset of symptoms with the turn of the year from 2017 to 2018 as cut-off (n = 47 vs. n = 39 patients with symptom onset before vs. since January 2018) (S1 Fig). Because of the shorter follow-up of the patients with onset of symptoms since 2018, patients with diagnostic delay greater than 36 months and onset of symptoms before 2018 were censored at 36 months within the quantile regression. Both methods revealed a marked reduction of the diagnostic delay after 2018.

### Patient journey in amyloidosis

28.8% of symptomatic ATTR, but only 8.3% of symptomatic AL amyloidosis patients had received their diagnosis after two contacts with physicians. More than 5 physicians until receiving correct diagnosis had been contacted by 20.3% of symptomatic ATTR and 25.0% of symptomatic systemic AL amyloidosis patients, respectively (patients with incidental diagnosis were excluded from this analysis).

*At disease onset*, primary contacts of patients across all subtypes were GP (36.8%) and registered specialists (42.1%) (Table 5). The amyloidosis centre played no role at this point. ATTR-amyloidosis patients contacted specialists most frequently (47.5%), followed by GPs (32.2%). More than two-thirds of the specialists were cardiologists (70.0%). In contrast, almost 50% of the patients with AL amyloidosis first contacted their GP.

**Table 5:** Primary contact at different stages during patient journey in amyloidosis in the total cohort and broken down for AL and ATTR amyloidosis.

Primary contact		at onset of symptoms			at diagnosis			after diagnosis		
		*overall*	*ATTR*	*AL*	*overall*	*ATTR*	*AL*	*overall*	*ATTR*	*AL*
**setting**	*number*	*95*	*71*	*26*	*115*	*74*	*26*	*118*	*74*	*26*
	general practitioner	36.8%	32.2%	47.8%	0.0%	0.0%	0.0%	7.6%	10.8%	0.0%
	specialist	42.1%	47.5%	26.1%	14.8%	13.5%	15.4%	15.3%	13.5%	19.2%
	non-university hospital	13.7%	13.6%	17.4%	33.9%	31.1%	34.6%	4.2%	2.7%	3.8%
	university hospital	6.3%	6.8%	8.7%	24.3%	14.9%	42.3%	6.8%	2.7%	11.5%
	amyloidosis center	0.0%	0.0%	0.0%	27.0%	36.5%	7.7%	63.6%	66.2%	65.4%
	no comment	1.1%	0.0%	0.0%	0.0%	4.1%	0.0%	2.5%	4.1%	0.0%
**discipine** *	*number*	*97*	*51*	*13*	*86*	*46*	*24*	*33*	*16*	*9*
	cardiologist	49.2%	70.0%	8.3%	37.2%	58.7%	20.8%	36.4%	75.0%	0.0%
	nephrologist	6.8%	2.5%	25.0%	10.5%	0.0%	33.3%	12.1%	0.0%	33.3%
	hematologist	1.7%	0.0%	8.3%	12.8%	4.3%	20.8%	21.2%	0.0%	55.6%
	others	42.4%	27.5%	58.3%	34.9%	32.6%	20.8%	24.2%	12.5%	11.1%
	no comment	0.0%	0.0%	0.0%	4.7%	4.3%	4.2%	6.1%	12.5%	0.0%

* if not GP or amyloidosis centre

*The diagnosis* itself was usually made in a hospital setting such as in the context of non-university (33.9%) and university (24.3%) hospitals and the amyloidosis centre (27.0%). In ATTR amyloidosis, the amyloidosis centre (36.5%) and non-university hospitals (31.1%) played a leading role in the diagnosis, while in case of systemic AL amyloidosis, the role of the amyloidosis centre was secondary (7.7%) and the diagnosis was primarily established by non-university (34.6%), and university hospitals (42.3%).

During *follow-up after diagnosis*, up to about two thirds of patients rated subtype-spanning the amyloidosis centre as their primary contact. In addition, registered specialists played an important role. The ATTR amyloidosis patients were primarily cared for by cardiologists (75%) and the AL amyloidosis patients primarily by hematologists (55.6%) or nephrologists (33.3%).

*At the time point of the evaluation*, 95.8% of the patients were in contact with GPs, ATTR amyloidosis patients (97.3%) more than AL amyloidosis patients (88.5%). Local specialist medical care was involved by 82.2% of the total cohort. Psychological support was received by 6.8% of the total cohort, 2.7% of ATTR and 15.4% of AL amyloidosis patients. 91.5% of the total cohort did not consider it necessary. Pastoral care was used by 4.2% of patients and relaxation techniques were practiced by 34.7% of the patients. Support from clinical social services could only be evaluated by 7.6% of the respondents. Palliative medical care was not used at all and not considered to be necessary by 99.2% of the patients. Self-help groups were attended by 13.6% of those affected; difficult access to self-help groups was only stated by 3.4%, while 82.2% of patients did not deem them necessary. Physiotherapy and physical therapy were used by 30.5% of the patients, 11.9% described access as difficult, the majority (56.8%) did not consider them necessary. In addition, amyloidosis patients stated that they would like guided sports activities (18/34 additional free text responses), especially specialized amyloidosis sports groups, rehabilitation therapy, relaxation techniques such as yoga, meditation und breathing techniques, nutritional advice and “detoxification” under chemotherapy.

According to the evaluation, the regular check-ups at the amyloidosis centre give the patients in general the feeling of safety (75.4%) which was more pronounced in AL amyloidosis patients (84.6%), but 23.7% do not feel reassured by this. 2 patients (3.4%) stated that they even feel sicker as a result of the regular check-ups, whereas 94.9% of the patients ruled this out.

### Needs on the way to diagnosis or at diagnosis

From the perspective of the patients (total cohort), the following aspects were rated as very important on the way to diagnosis or at diagnosis: professional competence (99.2%), comprehensibility of information (97.5%), trusting relationship (98.3%), information about the disease at diagnosis (96.6%) and necessary examinations (94.1%), diagnostic investigations in the centre (93.2%), a fixed reference person on the part of the doctor (93.2%), scope of information (94.1%), comprehensiveness of diagnostic investigations (91.5%), accessibility of the centre (92.4%), central contact person for organizational matters (82.2%), background information about the suspected disease (79.7%) and acceleration of the diagnosis (69.5%). Psychological support was not deemed important by 87.3% of the patients, but only 76.9% of AL amyloidosis patients. A fixed reference person on the part of the nursing staff (positive and negative rated in 49.2% each) and exchange about the disease with other affected persons (negative 62.7% and positive 36.4%) was perceived ambivalently. Especially in AL amyloidosis patients, the last two points were more important compared to the average.

### Preferred contact persons on the way to diagnosis, at diagnosis and in the course of treatment

**Fig 1:**
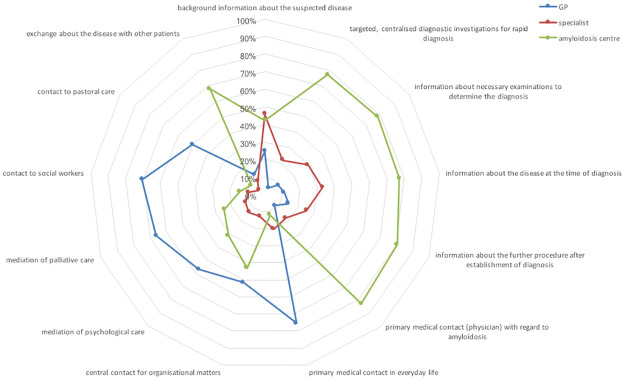
Preferred responsibilities on the way to or at diagnosis from the patients’ perspective. *On the way to diagnosis*, GPs are assigned a central role as the primary medical contact in everyday life as well as the central contact for organizational matters. The primary tasks of the amyloidosis centre are targeted, centralized diagnostic investigations for rapid diagnosis, information about the disease at the time of diagnosis as well as the necessary examinations to determine the diagnosis and the further procedure as well as primary medical contact (physician) with regard to the disease. Specialists play an important role in providing background information about the suspected disease, which is also seen as a task of the amyloidosis centre and to a lower extent of the GPs. The spectrum of tasks attributed to the specialists is similar to those of the amyloidosis centre. Subtype-specific analysis revealed no differences in the attributed responsibilities between ATTR and AL amyloidosis.

**Fig 2:**
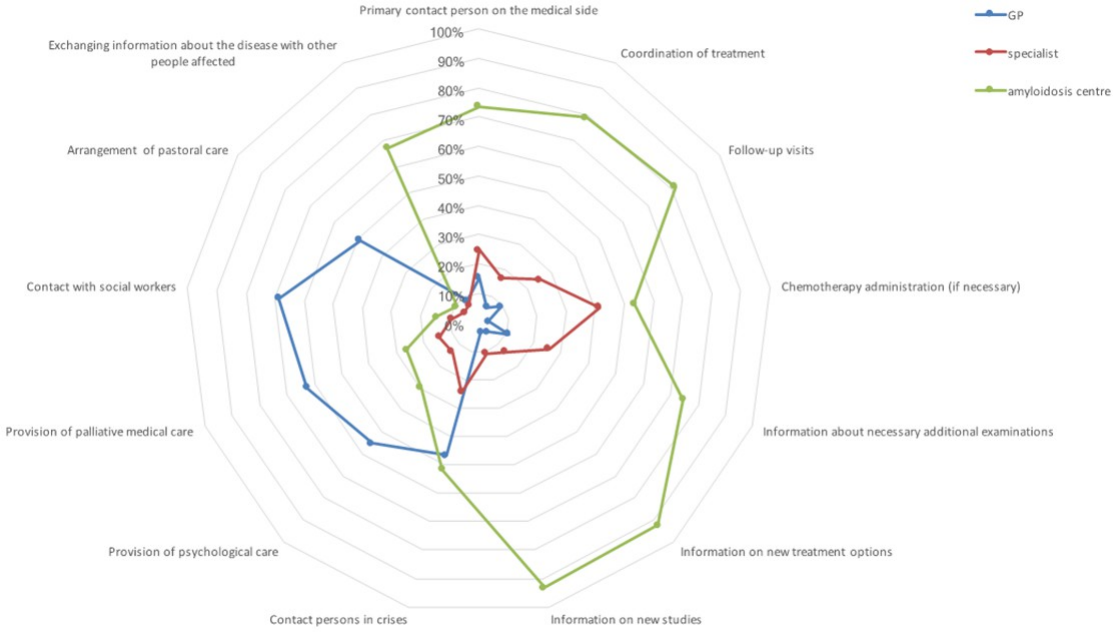
Preferred responsibilities during the further disease course from the patients’ perspective. *In the course of treatment*, patients see their GPs as the leading contact during crises in addition to the amyloidosis centre, which is primarily considered to be the contact and coordinator of treatment in this phase. Patients primarily wish to have their progress monitored as well as their need for information regarding necessary additional examinations, new treatment options and new studies fulfilled by the amyloidosis centre. Also in the further course of treatment, the profile of the tasks assigned to the specialists from the patient’s point of view essentially corresponds to those of the amyloidosis centre, even if the majority of patients attribute them to the amyloidosis centre. If chemotherapy is required, 41.5% would like to receive the administration by the local specialist. The mediation of psychological care, palliative medical connection as well as contact to social workers and pastoral care should primarily be mediated by GPs both on the way to diagnosis as well as at diagnosis and in the further course of treatment. In addition, some of the patients see the provision of psychological care, palliative care and contact with social workers as part of the tasks of the centre during all phases of the disease. Similar results are found in the subtype-specific analysis with the exception of the contact person during crises—AL amyloidosis patients see the responsibility primarily with the amyloidosis centre, while ATTR amyloidosis patients attribute this primarily to the GP.

The preferred contact persons from the patients’ point of view varied within the different phases in the course of the disease. *On the way to diagnosis* (Fig 1), the amyloidosis centre was seen as the primary medical contact for the disease and for rapid diagnosis. The GP should act as an important primary contact in everyday life and for organizational matters, while the specialist is deemed responsible for providing background information. *In the course of treatment* (Fig 2), the amyloidosis centre is primarily considered to be central medical contact and treatment coordinator. GPs should be the leading contact during crises according to ATTR amyloidosis patients whereas AL amyloidosis patients prefer the amyloidosis centre. If chemotherapy is required, 41.5% would like to receive the administration by the local specialist.

### Potential for optimization in the interaction between the amyloidosis centre and HCPs as well as with patients

An improvement in the exchange of information between HCPs and the amyloidosis centre by means of electronic medical records, directly transmitted short reports or via app with GPs and specialists was supported by the majority of patients (all ≥89%, S1 Table). Likewise, a hotline as well as information material for physicians would be desirable. A centre homepage with information material on the disease and studies was also advocated.

Patients would welcome an emergency hotline (89.9%) and telephone telemonitoring by specially trained nursing staff (81.1%). Overall, 66.9% of patients stated that they would make use of such a telemonitoring, 13.6% were unsure whether they would use it and 32.2% declined it. A telephone-based form of telemonitoring would be preferred to a Skype-based variant.

Overall, telemonitoring was better accepted in AL than ATTR amyloidosis patients. A subtype-specific analysis showed that only 19.2% of AL amyloidosis patients would decline telemonitoring, in contrast to nearly twice as many ATTR amyloidosis patients (37.8%, p = 0.055). The telephone-based variant would be used in 57.7% of AL compared to 45.9% in ATTR amyloidosis patients (p = 0.33) and the Skype-based variant by 38.5% of AL compared to 13.5% of ATTR amyloidosis patients (p = 0.02). An app for direct doctor-patient exchange was viewed ambivalently by the patients.

Information events (67.8%) as well as information material on the homepage (73.7%), in digital (72.9%) and printed form (71.2%) were advocated.

### Perceived limitations of the quality of life as consequence of the diagnosis “amyloidosis”

Overall, 56.8% of the patients reported feeling that their quality of life is fairly or very impaired by the amyloidosis disease, only 16.1% clearly denied this. In ATTR amyloidosis quality of life impairment seems to be more pronounced than in AL amyloidosis (AL 46.2% vs. ATTR 66.2%, p = 0.052). Quality-of-life restrictions due to fear of progression were described by 16.1% of patients, even though 56.8% denied this at all.

Increased anxiety since the onset of amyloidosis-related symptoms was described by 31.4% of the patients in varying degrees with emphasis on the AL amyloidosis patients (AL total 46.2%; ATTR total 31.1%), 65.3% of the patients excluded such feelings. A reduced quality of life due to anxiety was reported by about one third of the patients to varying degrees (total 28.8%) without subtype-specific differences. An anxiety disorder was documented in five (4.2%) of the total cohort. Anti-anxiety medication was taken by two of the five patients with known anxiety disorder, while only one patient reported psychological treatment. In contrast, a depressed mood since the onset of amyloidosis-related symptoms was stated or at least considered by 44.1% of the surveyed amyloidosis patients with similar results in the AL and ATTR amyloidosis subgroup. 51.7% excluded a depressed mood as a result of the diagnosis of amyloidosis. Depression was documented in seven patients (5.9%), specific drugs in two and psychotherapy in no case. The diagnosis depression was already made in five of the seven patients before the diagnosis of amyloidosis.

Of those who were still employed (n = 18), no patient was sure that they would be able to work until retirement age. Overall, two thirds of the surveyed patients saw their ability to work threatened by their current state of health, 27.8% ruled this out. However, only 22.2% were considering applying for early retirement for health reasons and 72.2% denied the question.

### Formulation of needs

Overall, 58.3% of the surveyed amyloidosis patients had talked in general about their worries in the context of outpatient care services (general practitioners, specialists) and in 83.1% with family members or friends (S2 Table). In the subtype-specific analysis, AL and ATTR amyloidosis patients talked to health care providers to a similar extent (AL vs. ATTR 57.7 vs. 60.8%, p = 0.622), but communication of needs with relatives and friends was significantly higher in AL amyloidosis (AL vs. ATTR 78.4 vs. 96.2%, p = 0.018).

In the next step, we tried to get a better understanding of the topics patients talk about and which aspects have not been discussed for what reason. The following topics were chosen: fear of a relapse and of the further course of the disease, dealing with death, physical changes and physical consequences of treatment, effects of the disease on sexuality and partnership, problems in the family or at work, fear for the professional future, financial security and advance care planning/ patient directive.

The most frequently discussed topic was advance directives and advance care planning (51.6%) followed by physical changes and physical consequences of treatment (37.3%). Approximately one third of patients communicated about dealing with death (29.4%) as well as the fear of the future course (30.1%). Nearly every 5th patient articulated fear of a relapse (22.2%), the effects of the disease on sexuality (20.9%) and partnership (20.9%). Patients with AL amyloidosis discussed the mentioned topics more frequently compared to ATTR amyloidosis patients except for fear of the further disease course and effects of the disease on sexuality.

Less frequently discussed topics included financial security (16.3%), problems in the family environment (11.1%), fear concerning their professional future (4.6%) and problems at work (2.6%) in the total cohort.

Among the 18 patients still employed, financial security was discussed in 44.4%, problems at work in 22.2% and fear for the professional future in 22.2%.

Concerns that were not discussed despite the need for discussion were topics of advance care planning (18.3%), physical changes and consequences of treatment (7.8%), fear of the further disease course (5.9%) and relapse (3.9%) as well as the effects of the disease on sexuality (3.9%) and partnership (0.7%) and dealing with death (2.0%). Item-non-response was 0.7–2.0%.

## Discussion

Systemic amyloidosis represents a life-threating disorder which can be associated with high psychosocial disease burden. Up to now, there have been no specific data on the care situation of amyloidosis patients in Germany. Furthermore, the specific needs of amyloidosis patients are only partially understood and have not been systematically analyzed across subtypes yet [19, 23].

The _A_MY-NEED_S_ research program aims to systematically assess the current state of care for amyloidosis patients and unmet needs with the long-term goal of implementing a disease-specific care concept in the German health care system.

Therefore, we outlined the patient journey and analyzed the diagnostic delay:

About one in five patients received the diagnosis of amyloidosis as an incidental finding without having noticed any subjective symptoms primarily in the clinic setting. The majority of these patients was diagnosed in the recent past, so that this may be explained (at least partly) by the use of advanced technical facilities such as strain analysis among echocardiography, newly established non-invasive diagnostic tools e.g. implementation of bone scintigraphy according to Gillmore et al. [9] and increased awareness of amyloidosis as consequence of congress activities, training events and the establishment of the local amyloidosis centre. However, this is also consistent with results from a prevalence study in which 21.7% of the identified amyloidosis patients were asymptomatic [24]. The high percentage of incidental findings in the clinic setting might be also a result of the different distribution of tasks and offered diagnostic investigations. Endomyocardial biopsies, for example, are only performed in the clinic setting.

The diagnostic delay in our sample of 7.9–9.0 (AL 6.0; ATTR 12.0) months is in line with the diagnostic delay observed in another German cohort of only AL amyloidosis patients of 7.0 months [6]. However, compared to international data [3–5], the diagnostic delay in German patients is much smaller. This positive trend can also be seen in the number of physicians contacted—the cohort reported here only contacted 5 or more physicians in 23.2% compared to up to 50% of patients according to Hester et al. 2021.

A possible explanation could be the growing general awareness among HCPs due to frequent discussions about amyloidosis at recent congresses as well as information material and information events for local HCPs by the pharmaceutical industry because of newly approved drugs in analogy to the British observations [8].

As the primary contact at diagnosis is in the majority of patients not the amyloidosis centre, there could be also an indirect effect by upcoming amyloidosis centres and outpatient departments resulting in few reluctance on the part of local HCPs to initiate specific diagnostic investigations because of easy and low-threshold access. This is in line with the observation that the diagnostic delay is smaller in the city and the district of Würzburg than outside without reaching significance (median 6 (city) vs. 7 (district) vs. 10.5 months (outside)).

About half of the surveyed patients reported a reduction in their quality of life by amyloidosis. One third claimed to suffer from feelings of anxiety. Depressed mood since the onset of amyloidosis-related symptoms was reported or considered by 44.1% of patients with similar results in the subtype-specific analysis. Overall, these results are comparable to results from other surveys such as Shu et al. in 1226 AL amyloidosis patients, who found evidence of depression in 37.0% and anxiety in 46.7% [25]. Additionally, heart failure is known to be associated with 3–5 times higher prevalence of depression resulting in prevalence rates of 20–40% [26, 27]. Moreover, the role of fatigue in this context is not clear yet. Systematic further investigations are required for a better understanding.

Prevalence of known depression in our cohort was similar compared to the results of German health survey DEGS1 published by Busch et al. 2013 [28]. In accordance with the DEGS1 results showing 10–40% of the patients with depression under treatment, only 20% of patients received psychotherapy [29].

This discrepancy between reported feelings of anxiety and depressed mood one the one hand and low prevalence of ICD-10 coded psychological disorders on the other hand, might be explained by underdiagnosis in the elderly [30, 31] maybe as consequence of a misinterpretation as aging phenomenon [32]. Another explanation could be (elder) patients’ fear of stigmatization.

The relatively high distress contrasts with the comparatively low need for communication.

As expected, patients talk about their needs primarily with their relatives and direct caregivers rather than with the local HCPs from whom they primarily receive care. The support from family members and the resulting burden for them have been described elsewhere [33, 34].

The discrepancy between our cohort and the themes reported by Magliano et al. of restrictions in leisure activities, fear of loss and worries about the future could be explained by the different stages of the disease [34]. While Magliano et al. exclusively interviewed symptomatic ATTRv amyloidosis patients, the cohort reported here is very heterogeneous with partly absent or minor symptoms. Most patients see no need for palliative care or psychological care. The extent to which this is due to denial or a lack of knowledge about the course of the disease cannot be estimated based on the available data. Nevertheless, advance care planning is a frequently discussed topic, as well as dealing with death, physical consequences of treatment and fear of the further disease course. However, ¼ of the patients could not address topics of end of life care and advance directives, even if they would like to. This need could easily be met by implementing the already existing conversation concepts, including corresponding conversation guides, into the routine and could be supported by specially trained nurses and social workers in close interaction with involved physicians [35].

It is noticeable that patients primarily see the referral to social workers as well as any necessary psychological and palliative medical connection as the responsibility of the general practitioner. Overall, this indicates a low level of knowledge about the health care system in terms of navigational health literacy among the patients concerned and the need of information, contact points and case management. It is conceivable that the attribution to the GP’s area of responsibility has to be interpreted as a reaction of helplessness, since in the German health system the GP is nearly always the first point of contact and often a person of trust. Individuals with at least one chronic condition or long-standing health problem are known to have a percentage of low navigational health literacy of 86.2% [36].

The otherwise observed shift in responsibilities from the patients’ point of view seems to be expected and characteristic for the German health care system:

On the way to diagnosis, GPs and local specialists are primary contacts as expected from the structure of the German health care system [18], especially cardiologists in the case of ATTR amyloidosis.

Moreover, the need for increased awareness and specific information of this group of physicians according to Harris et al. [37] and Mircsof et al. [38] has to be emphasized.

The patients’ preference of the amyloidosis centre in a pivotal role after diagnosis with coordination of treatment is in line with the “ideal” amyloidosis programme according to Nativi-Nicolau et al. as one that provides physicians with expertise in ATTR-CM, sufficient time for patients and relatives, comprehensive patient care, and opportunities to participate in research/clinical trials [39]. As >80% of patients were in contact with GPs and outpatient specialists at the time point of evaluation during follow-up at the local amyloidosis centre, a comprehensive patient care makes a well-functioning interface between so many involved interdisciplinary HCPs mandatory. An adequate communication and information transfer appears challenging against the backdrop of time and cost pressure in the current health care system. Specific concepts are needed to overcome the communication gap. According to the survey results, specially tailored telemonitoring concepts combining a telephone-based approach and direct interaction with specially trained nurses as communicators between centre, patient and peripheral HCPs might be a realistic and cost effective approach. Beyond timely transfer of information, continuous symptom control by adequate control of volume status in case of heart failure and dose adjustment of analgetics may result in better quality of life and probably improved prognosis. The patients’ openness towards telephone-based, but not app- or video-based telemonitoring concepts, might be explained by age. The feasibility of telemonitoring concepts have already proven successfully for symptom complexes as heart failure [40–43], as well as in the rare disease area [44, 45] and in general during COVID pandemic.

Strengths of the proposed project are the innovative and well-structured design with inclusion of several subgroups and depiction of several disease stages within the patients, although depiction of needs among end-stage patients is lacking. Additionally, we were able to reach a remarkable case number for a monocentric approach within the German health care system with currently scarce data, which is, however, comparatively low in the international comparison with centre-independent surveys [19] and network studies [46]. Of course, the monocentric approach has to be mentioned as limitation. The approach of including patients of all subtypes and manifestation patterns, e.g. cardiac and non-cardiac involvement, is both advantageous and disadvantageous. On the one hand, it obviously results in a very heterogeneous patient group. On the other hand, the approach results in a real-world cohort that also takes into account rarer entities and thus does not only represent the frequently investigated majority. Further, a retrospective approach was chosen for estimation of the diagnostic delay, but validity of results was checked by comparison with data extracted from medical documentation to minimize bias.

## Conclusion

The diagnostic delay was comparatively low in this sample exemplifying the German health care system, with indications for improvement in the recent past. Detailed characterization of patient journey, contact partners, and so far met and unmet needs at different disease stages in amyloidosis allow for a deeper understanding. Advance care planning is a relevant, yet only partly met need among this patient population and should be addressed systematically by transfer of already established conversation guides. The patient-preferred coordination of treatment by the centre, the involved comprehensive care, and the variety of involved HCPs requires a well-functioning network which is not yet existent. Therefore, a disease-specific comprehensive care concept with the highly visible offer of social work support as well as psychological and palliative care is required. Telemedical approaches in combination with classical and innovative communication methods between all key players might be promising.

## Supporting information

S1 Fig Diagnostic delay over time. To estimate the effect of the key changes such as increasing establishment of specialized outpatient departments and amyloidosis centre(s), establishment of non-invasive diagnosis of cardiac ATTR amyloidosis by bone scintigraphy, increasing availability of new drugs and growing disease awareness among HCPs, we i) analyzed the diagnostic delay year by year and ii) performed a quantile regression analysis regarding the diagnostic delay depending on the onset of symptoms with the turn of the year from 2017 to 2018 as cut-off (n = 47 vs. n = 39 patients with symptom onset before vs. since January 2018). Patients with diagnostic delay greater than 36 months and onset of symptoms before 2018 were censured at 36 months because of the shorter follow-up of patients with symptom onset after 2018. The diagnostic delay shows a clear decline in the recent past in the annual breakdown (i). Quantile regression analysis (ii) shows significant improvement of diagnostic delay across all quantils comparing diagnostic delay with symptom onset bevor vs. since January 2018. The extent of delay reduction is quantified by the included numeric values [months]. i) Annual breakdown of diagnostic delay depending on time point of onset of symptoms. ii) Quantile regression.
(DOCX)

S1 Table Evaluation of possible optimization approaches by the patients. (DOCX)

S2 Table Discussion about needs. (DOCX)

S1 File (PDF)

## Abbreviations

AA: (Apo)serum AA
AL (amyloidosis): light chain (amyloidosis)
AmyKoS: amyloidosis cohort study of the Interdiscplinary Amyloidosis Centre of Northern Bavaria
ANS: autonomous nervous system
ApoC2 (amyloidosis): Apolipoprotein C2 (amyloidosis)
ATTR (amyloidosis): transthyretin (amyloidosis)
ATTRv amyloidosis: hereditary transthyretin amyloidosis
ATTRwt amyloidosis: wild-type transthyretin amyloidosis
CA: cardiac amyloidosis
CCI: Charlson comorbidity index
CRF: case report form
DEGS1: German health survey called DEGS1
ECOG: Eastern Co-operative Oncology Group Performance Status
GIT: gastrointestinal tract
GP: general practitioners
HCP: health care provider
ICD-10: ICD-10 diagnosis code
NT-proBNP: N-terminal pro B-type natriuretic peptide
PNS: peripheral nervous system
