# Supplementary material for: Single German centre experience with patient journey and care-relevant needs in amyloidosis: The German AMY-NEEDS research and care program
Source: PLoS One. 2024 May 20;19(5):e0297182. doi: 10.1371/journal.pone.0297182 (PMC11104610; doi:10.1371/journal.pone.0297182)
Supplement: S1 Fig — To estimate the effect of the key changes such as increasing establishment of specialized outpatient departments and amyloidosis centre(s), establishment of non-invasive diagnosis of cardiac ATTR amyloidosis by bone scintigraphy, increasing availability of new drugs and growing disease awareness among HCPs, we i) analyzed the diagnostic delay year by year and ii) performed a quantile regression analysis regarding the diagnostic delay depending on the onset of symptoms with the turn of the year from 2017 to 2018 as cut-off (n = 47 vs. n = 39 patients with symptom onset before vs. since January 2018). Patients with diagnostic delay greater than 36 months and onset of symptoms before 2018 were censured at 36 months because of the shorter follow-up of patients with symptom onset after 2018. The diagnostic delay shows a clear decline in the recent past in the annual breakdown (i). Quantile regression analysis (ii) shows significant improvement of diagnostic delay across all quantils comparing diagnostic delay with symptom onset bevor vs. since January 2018. The extent of delay reduction is quantified by the included numeric values [months]. i) Annual breakdown of diagnostic delay depending on time point of onset of symptoms. ii) Quantile regression. (DOCX) [file pone.0297182.s001.docx]

| **year of onset of symptoms** | **median** | **25% percentile** | **75% percentile** | **n** |
| --- | --- | --- | --- | --- |
| 2003 | 120.0 | 120.0 | 120.0 | 2 |
| 2006 | 24.0 | 24.0 | 24.0 | 1 |
| 2009 | 36.0 | 36.0 | 36.0 | 1 |
| 2010 | 72.0 | 72.0 | 72.0 | 1 |
| 2012 | 60.0 | 60.0 | 60.0 | 2 |
| 2013 | 21.0 | 4.5 | 51.0 | 7 |
| 2014 | 60.0 | 36.0 | 60.0 | 5 |
| 2015 | 9.0 | 7.0 | 13.5 | 3 |
| 2016 | 15.0 | 5.5 | 34.5 | 17 |
| 2017 | 6.5 | 1.3 | 24.0 | 17 |
| 2018 | 3.0 | 2.3 | 5.3 | 7 |
| 2019 | 3.0 | 1.0 | 6.3 | 16 |
| 2020 | 3.0 | 2.0 | 10.3 | 12 |
