# Supplementary material for: Single German centre experience with patient journey and care-relevant needs in amyloidosis: The German AMY-NEEDS research and care program
Source: PLoS One. 2024 May 20;19(5):e0297182. doi: 10.1371/journal.pone.0297182 (PMC11104610; doi:10.1371/journal.pone.0297182)
Supplement: S1 Table — (DOCX) [file pone.0297182.s002.docx]

*Supplementary Table 1: Evaluation of possible optimization approaches by the patients*

| **Approaches for optimization** | **overall (n=118)** | | | | |  |
| --- | --- | --- | --- | --- | --- | --- |
|  | ***yes, definitely*** | ***possibly*** | ***no*** | ***no answer*** |  |  |
|  |  |  |  |  |  | |
| electronic medical records | *67.8%* | *21.2%* | *8.5%* | *2.5%* |  | |
| directly transmitted short reports | *88.1%* | *7.6%* | *7.6%* | *2.5%* |  | |
| hotline for peripheral HCPs | *76.3%* | *13.6%* | *7.6%* | *2.5%* |  | |
| information material for peripheral HCPs | *71.2%* | *20.3%* | *5.9%* | *2.5%* |  | |
| app for interaction with GPs and specialists | *64.4%* | *24.6%* | *9.3%* | *1.7%* |  | |
| homepage with informations about disease, trials, … | *80.5%* | *11.9%* | *5.1%* | *2.5%* |  | |
| emergency hotline | 78.0% | 11.9% | 9.3% | 0.8% |  | |
| telephone-based telemonitoring by amyloidosis or heart failure nurses | 57.6% | 25.4% | 15.3% | 1.7% |  | |
| app for doctor-patient interaction | 33.1% | 20.3% | 45.8% | 0.8% |  | |
| app for information exchange between doctor and patient | 30.5% | 21.2% | 47.5% | 0.8% |  | |
| information events | 49.2% | 18.6% | 30.5% | 1.7% |  | |
| information material on the homepage | 63.6% | 10.2% | 24.6% | 1.7% |  | |
| digital information material | 61.0% | 11.9% | 26.3% | 0.8% |  | |
| printed information brochures | 63.6% | 7.6% | 28.0% | 0.8% |  | |
