# Supplementary material for: Single German centre experience with patient journey and care-relevant needs in amyloidosis: The German AMY-NEEDS research and care program
Source: PLoS One. 2024 May 20;19(5):e0297182. doi: 10.1371/journal.pone.0297182 (PMC11104610; doi:10.1371/journal.pone.0297182)
Supplement: S2 Table — (DOCX) [file pone.0297182.s003.docx]

*Supplementary Table 2: Discussion about needs*

*a) Conversation about worries in accordance with patients’ needs*

| **Conversation about worries in accordance with their needs** | **overall (n=118)** | | | **ATTR (n=74)** | | | **AL (n=26)** | | |
| --- | --- | --- | --- | --- | --- | --- | --- | --- | --- |
|  | yes | no | no  answer | yes | no | no  answer | yes | no | no  answer |
| with outpatient care services such as GPs and specialists on site | 58.5% | 38.1% | 3.4% | 60.8% | 35.1% | 4.1% | 57.7% | 42.3% | 0% |
| with family, friends, etc. | 83.1% | 13.6% | 3.4% | 78.4% | 17.6% | 4.1% | 96.2% | 3.8% | 0% |
| no, but there is a need and the desire to talk | 1.7% | 94.9% | 3.4% | 1.4% | 94.6% | 4.1% | 0% | 96.2% | 0% |
| no, despite need, but without desire to talk | 3.4% | 93.2% | 3.4% | 4.1% | 91.9% | 4.1% | 0% | 100.0% | 0% |
| no because of lacking need to talk | 11.0% | 85.6% | 3.4% | 14.9% | 81.1% | 4.1% | 0% | 100.0% | 0% |

*b) Topics*

| **Topic** | **discussed** | **not discussed** | | | **no**  **comment** |
| --- | --- | --- | --- | --- | --- |
|  |  | *but wanted* | *unwanted* | *not necessary* |  |
| Advance care planning | 51.6% | 18.3% | 5.9% | 72.5% | 2.0% |
| Physical changes & consequences of treatment | 37.3% | 7.8% | 3.3% | 71.2% | 0% |
| Fear of the future course | 30.1% | 5.9% | 2.0% | 64.7% | 0% |
| Dealing with death | 29.4% | 3.9% | 1.3% | 59.5% | 0.7% |
| Fear of relapse | 22.2% | 3.9% | 1.3% | 54.9% | 0.7% |
| Effects of the disease on sexuality | 20.9% | 2.0% | 0.7% | 51.0% | 0% |
| Effects of the disease on partnership | 20.9% | 0.7% | 0.7% | 47.7% | 0% |
| Financial security | 16.3% | 0.7% | 0.7% | 39.9% | 0.7% |
| Problems in the family | 11.1% | 0% | 0.7% | 37.9% | 0.7% |
| Fear for professional future | 4.6% | 0% | 0.7% | 30.7% | 0.7% |
| Problems at work | 2.6% | 0% | 0.7% | 4.6% | 0.7% |
