# Supplementary material for: Single German centre experience with patient journey and care-relevant needs in amyloidosis: The German AMY-NEEDS research and care program
Source: PLoS One. 2024 May 20;19(5):e0297182. doi: 10.1371/journal.pone.0297182 (PMC11104610; doi:10.1371/journal.pone.0297182)
Supplement: S1 File — (PDF) [file pone.0297182.s004.pdf]

General information

Date __/__/20__
T T M M J J J J

Please state your gender O male O female

How old are you? ___ years

Please state your nationality: _______________

What is the distance between your home and the amyloidosis centre?
O City of Würzburg O County of Würzburg
O 0-50 km O 50-100 km O 100-150 km O >150 km

How old were you when you were diagnosed? ___ years

What was the time span between the first symptoms and diagnosis?
__ years or __ months

Which first complaints did you notice? (max. 3 keywords)

How many different doctors did you contact before you received a correct (?) diagnosis?
O 1 O 2 O 3 O 4 O 5 and more

Who was/is your primary contact
- after the onset of the first symptoms until diagnosis?
O general practitioner (GP)
O specialist in private practice
if yes: O cardiologist O nephrologist O hematologist
O other specialist: ____________________________
O hospital doctor (specialization: ________________)
O University Hospital (specialization: _____________)

- at the time of diagnosis or who made the diagnosis?
O general practitioner (GP)
O specialist in private practice
if yes: O cardiologist O Nephrologist O hematologist
O other specialist: ____________________________
O hospital doctor (specialization: ________________)
O University Hospital (specialization: _____________) O Amyloidosis Center

- after diagnosis?
O general practitioner (GP)
O specialist in private practice
if yes: O cardiologist O nephrologist O hematologist
O other specialist: ____________________________
O hospital doctor (specialization: ________________)
O University Hospital (specialization: _____________)

Questions about you as person

What is your marital status?

O single
O married
O marriage-like partnership
O divorced / separated
O widowed

Do you live with a permanent partner?

O yes
O no

Do you have children?

O yes
O no

Do you have grandchildren?

O yes
O no

What school education did you receive?

O secondary school
O middle school
O polytechnic secondary school
O advanced technical college certificate
O A-levels
O no school-leaving qualification
O other: ____________________________

What kind of vocational training do you have?

O Apprenticeship (vocational-in-company training)
O Technical school
(master craftsman school, technical school, vocational academy, technical academy)
O University of Applied Sciences, School of Engineering
O University, college
O no professional training
O other vocational training: ____________________________

Are you currently employed?

O yes, full-time
O yes, at least half-time
O yes, less than half-time
O no, in training
O no, unemployed / unemployable
O no, disability pension
O no, old-age pension
O no, other ______________________

In which professional position are you mainly employed at present?

O worker
O employee
O civil servant
O self-employed
O pensioner
O other _______________

Social demographic data

Which profession do you have or have you had? (keyword)

What is the total monthly net income of your household?
(Net income means the sum of wages/salary/income etc., in each case after deduction of the taxes and social security contributions).

O less than 500 euros
O 500 to under 1,000 euros
O 1,000 to under 1,500 euros
O 1,500 to under 2,000 euros
O 2,000 to under 2,500 euros
O 2,500 to under 3,000 euros
O 3,000 to under 3,500 euros
O 3,500 euros and more

Do you have a reduced earning capacity pension?

O yes   O no   O applied for

If yes, what is the extent of the reduction in earning capacity?
O total reduction in earning capacity
O partial reduction in earning capacity
→ How many hours do you work per day? ____ hours

Are you currently on sick leave?

O yes O no

How long have you been on sick leave since diagnosis?

O______% of the time since diagnosis on sick leave

O no sick leave

Do you use a severely disabled person's card?

O no

O applied for

O yes → with what degree of disability? _________ %

Do you have a care degree?

O no

O applied for

O yes → which one? _________

Do you regularly need help from third parties for everyday activities?

O yes O no

If so:

For which activities?

O body care

O household activities

O shopping

O other: _________________________

Who primarily supports you?

O spouse/life partner

O children/grandchildren

O other relatives

O friends

O nursing service

O domestic help

O others: ________________

If you are currently already in retirement, please skip the next 3 questions.

Thinking about your current state of health and professional capacity, do you think you can work until you reach retirement age? (Please select only one option)

O unsure O rather not O no way

Do you consider your current state of health to be a permanent threat to your ability to work?

O yes O no

Are you currently thinking about applying for a pension (early retirement for health reasons)?

O yes O no

Disease-specific situation

How would you describe the clinical picture of "amyloidosis" to a stranger in a few words?

Which form of amyloidosis do you have?

- O light chain amyloidosis (AL amyloidosis)
- O transthyretin amyloidosis (ATTR amyloidosis)
  - O wild-type (age-related form, not hereditary)
  - O genetic form (hereditary)
- O AA amyloidosis
- O other form, namely ___________________
- O I do not know

Which organs are affected in your case? (multiple answers possible)

- O heart
- O kidney
- O gastrointestinal tract
- O liver
- O soft tissues
- O nervous system
- O others, namely ___________________
- O I do not know

Are you currently receiving treatment for amyloidosis? O yes O no

If yes: Which kind of treatment are you currently receiving? (multiple selection possible)

- O chemotherapy
- O radiation therapy
- O TTR stabilizer (tafamidis meglumine)
- O gene silencer (patisiran, inotersen)
- O study drug as part of the ______________________ study
- O other: ___________________

Have you received treatments in the past? O yes O no

If yes: Which kind of treatment? (multiple selection possible)

- O chemotherapy
- O radiation therapy
- O TTR stabilizer (tafamidis meglumin)
- O gene silencer (patisiran, inotersen)
- O study drug
- O other: ___________________

What is your disease situation?

O first diagnosis   O no activity   O stable disease
O relapse   O progression   O I do not know

Do you consider your disease situation to be advanced?

O yes   O no   O I do not know

Self-assessment - general situation

I feel limited in my quality of life because of the amyloidosis disease.
O not at all O a little O fairly O a lot

I feel limited in my quality of life due to the treatment of the amyloidosis disease.
O not at all O a little O fairly O a lot

The regular check-ups at the centre give me a sense of security.
O not at all O a little O fairly O a lot

The regular check-ups at the centre make me feel sicker.
O not at all O a little O fairly O a lot

Since the onset of amyloidosis, I feel more tired.
O not at all O a little O fairly O a lot

Shortness of breath is a relevant problem for me.
O not at all O a little O fairly O a lot

The fear that the disease could progress limits my quality of life.
O not at all O a little O fairly O a lot

Since the onset of the amyloidosis-related symptoms, I have had increased anxiety.
O not at all O a little O fairly O a lot

I have the impression that anxiety affects my quality of life.
O not at all O a little O fairly O a lot

Is an anxiety disorder known/diagnosed?
O yes O no
If yes:
Do you regularly take anxiety-relieving medication for this? O yes O no
If yes, which ones? ______________________________
Are you receiving psychological/psychiatric treatment for this? O yes O no
Did the anxiety disorder already exist before amyloidosis diagnosis? O yes O no

Since the onset of the amyloidosis-related symptoms, I have had more periods of depressed mood, which affects my quality of life.
O yes O possibly O no O no information

Is a depression disorder known/diagnosed?
O yes O no
If yes:
Do you regularly take medication for this? O yes O no
If yes, which ones? ______________________________
Are you receiving psychological/psychiatric treatment for this? O yes O no
Did the depression already exist before the amyloidosis diagnosis? O yes O no

Recording the current situation

Since receiving the diagnosis of amyloidosis, have you talked about your concerns and stresses related to the disease according to your needs?
(multiple selection possible)

- O yes, within the framework of outpatient care services such as general practitioners and specialists on site
- O yes, with my family, my friends, etc.
- O no, but I would like to talk about it
- O no, I don't want to talk about it
- O no, I do not have such a need

Do you currently use or have you used one or more of the following care services?
If no, please indicate if you would like to use it but have no or difficult access to it, or if you do not consider it necessary.

	**yes,** I make use of	**no,** access difficult	**no,** not necessary
GP care	O	O	O
Specialist care	O	O	O
Psychotherapy/psychological counselling	O	O	O
Self-help group	O	O	O
Physiotherapy	O	O	O
Domestic help	O	O	O
Palliative care	O	O	O
Pastoral care	O	O	O
Social service	O	O	O
Learning relaxation techniques	O	O	O
Other follow-up care (e.g. sports)	O	O	O

What other care services have you used or would you like to use? Please list keywords.

Needs ON THE WAY TO or AT DIAGNOSIS

How relevant are the following points for you **on the way to diagnosis or at diagnosis**:

	not at all	little	moderately	a lot
accelerating the diagnosis	O	O	O	O
centralised diagnostics in the centre	O	O	O	O
extensiveness of the diagnostics	O	O	O	O
information about necessary examinations	O	O	O	O
preliminary information about the suspected disease (before diagnosis)	O	O	O	O
information about the disease at diagnosis	O	O	O	O
comprehensibility of the information	O	O	O	O
scope of information	O	O	O	O
fixed reference person on the part of the doctor	O	O	O	O
trusting relationship	O	O	O	O
professional competence	O	O	O	O
accessibility	O	O	O	O
fixed reference person on the part of the nursing staff	O	O	O	O
central contact person for organizational matters	O	O	O	O
sharing about the disease with others affected	O	O	O	O
psychological care	O	O	O	O

Additions:

Which of the above points do you think are already fulfilled in the amyloidosis centre? Which less?

	not at all	little	moderately	good
acceleration of the diagnosis compared to diagnostic investigations by GP or specialist	O	O	O	O
compact diagnostics at the centre from a single provider	O	O	O	O
extensiveness of the diagnostics	O	O	O	O
information about necessary examinations	O	O	O	O
preliminary information about the suspected disease (before diagnosis)	O	O	O	O
information about the disease at diagnosis	O	O	O	O
comprehensibility of the information	O	O	O	O
scope of information	O	O	O	O
fixed reference person on the medical side	O	O	O	O
trusting relationship	O	O	O	O
professional competence	O	O	O	O
accessibility	O	O	O	O
fixed reference person on the part of the nursing staff	O	O	O	O
central contact person for organizational matters	O	O	O	O
sharing about the disease with others affected	O	O	O	O
psychological care	O	O	O	O

Additions:

Needs ON THE WAY TO or AT DIAGNOSIS

Who do you think should be responsible for the following tasks? (Multiple answers are possible)

	GP	specialist (on site)	amyloidosis centre
preliminary information about the suspected disease (before diagnosis)	O	O	O
compact (centralised) diagnostics for rapid diagnosis of suspected amyloidosis	O	O	O
information about the necessary examinations	O	O	O
information about the disease at diagnosis	O	O	O
discussion of the further procedure after diagnosis	O	O	O
primary contact person on the part of the doctor with regard to amyloidosis	O	O	O
primary medical contact in everyday life	O	O	O
central contact for organizational matters	O	O	O
arrangement of psychological support	O	O	O
arrangement of palliative care	O	O	O
contact with social workers	O	O	O
pastoral care	O	O	O
exchange about the disease with other patients	O	O	O

Additions:

Needs DURING THE TREATMENT PROCESS

Who do you think should be primarily responsible for the following tasks?
(Multiple answers are possible)

	GP	specialist (on site)	amyloidosis centre
primary contact on the medical side	O	O	O
coordination of treatment	O	O	O
follow-up	O	O	O
chemotherapy administration (if necessary)	O	O	O
information about necessary additional examinations	O	O	O
information on new treatment options	O	O	O
information on new trials	O	O	O
contact person in crises	O	O	O
arrangement of psychological care	O	O	O
arrangement of palliative care	O	O	O
contact with social workers	O	O	O
mediation of pastoral care	O	O	O
exchange about the disease with other people affected	O	O	O

Additions:

Needs DURING THE TREATMENT PROCESS

How could the cooperation between the amyloidosis centre and general practitioners or specialists in private practice be improved?

	in any case	possible	no
electronic health record	O	O	O
directly transmitted short reports	O	O	O
hotline for doctors	O	O	O
information material for doctors	O	O	O
app for exchanging information with GPs and specialists	O	O	O
centre homepage with information on the disease, trials, …	O	O	O

Additions:

What could improve patient care in terms of direct interaction between the centre and the patient?

	in any case	possible	no
emergency hotline	O	O	O
telemonitoring via telephone by specially trained amyloidosis or heart failure nurses	O	O	O
app for doctor-patient exchange	O	O	O
information events	O	O	O
What frequency would be desirable? ______ x/year?			
app for information exchange between doctor and patient	O	O	O
homepage with information material	O	O	O
digital information material	O	O	O
printed information brochures	O	O	O

Additions:

Do you use a smartphone regularly?
O yes O no

Would you use an app to send information to the doctor/amyloidosis centre?
O yes O possibly O no

Would you use telemonitoring by specially trained nursing staff? (multiple selection possible)
O yes, by phone O yes, via Skype O no O I don't know

Have you discussed or are you still discussing the following topics since you were diagnosed with amyloidosis?

	**yes,** I have discussed/ I discuss	**no,** but I would like to discuss	**no,** I would not like to discuss	**no,** not necessary
fear of relapse	O	O	O	O
fear of the further disease course	O	O	O	O
dealing with death	O	O	O	O
physical changes and consequences of treatment	O	O	O	O
effects of the disease on sexuality	O	O	O	O
effects of the disease on the partnership	O	O	O	O
problems in the family	O	O	O	O
problems at the workplace	O	O	O	O
fear for my vocational future	O	O	O	O
financial security	O	O	O	O
advance directive/advance care planning	O	O	O	O

Other topics:

Coping strategies

Often, distracting activities in addition to dealing directly with the disease can also help to cope with stress. Have you done the following **activities** since you were diagnosed with amyloidosis?

	not at all	little	moderately	a lot
physical activities (walking, cycling, football etc.)	O	O	O	O
social activities (meeting with friends or family etc.)	O	O	O	O
creative activities (painting, handicrafts, making music, etc.)	O	O	O	O
cultural activities (theatre, cinema or museum visit etc.)	O	O	O	O
relaxing activities (Yoga, autogenic training, etc.)	O	O	O	O
spiritual activities (meditation, prayer, etc.)	O	O	O	O
cosy activities (reading, watching TV, listening to music, etc.)	O	O	O	O

other activities:
